# Mantle Hg isotopic heterogeneity and evidence of oceanic Hg recycling into the mantle

**DOI:** 10.1038/s41467-022-28577-1

**Published:** 2022-02-17

**Authors:** Runsheng Yin, Di Chen, Xin Pan, Changzhou Deng, Liemeng Chen, Xieyan Song, Songyue Yu, Chuanwei Zhu, Xun Wei, Yue Xu, Xinbin Feng, Joel D. Blum, Bernd Lehmann

**Affiliations:** 1 State Key Laboratory of Ore Deposit Geochemistry, Institute of Geochemistry, Chinese Academy of Sciences, Guiyang, China; 2 University of Chinese Academy of Sciences, Beijing, China; 3 Second Institute of Oceanography, Ministry of Natural Resources, Hangzhou, China; 4 First Institute of Oceanography, Ministry of Natural Resources, Qingdao, China; 5 Laboratory for Marine Geology, Qingdao National Laboratory for Marine Science and Technology, Qingdao, China; 6 State Key Laboratory of Environmental Geochemistry, Institute of Geochemistry, Chinese Academy of Sciences, Guiyang, China; 7 Department of Earth and Environmental Sciences, University of Michigan, Ann Arbor, MI USA; 8 Mineral Resources, Technical University of Clausthal, Clausthal-Zellerfeld, Germany

**Keywords:** Geochemistry, Petrology

## Abstract

The geochemical cycle of mercury in Earth’s surface environment (atmosphere, hydrosphere, biosphere) has been extensively studied; however, the deep geological cycling of this element is less well known. Here we document distinct mass-independent mercury isotope fractionation (expressed as Δ^199^Hg) in island arc basalts and mid-ocean ridge basalts. Both rock groups show positive Δ^199^Hg values up to 0.34‰ and 0.22‰, respectively, which deviate from recent estimates of the primitive mantle (Δ^199^Hg: 0.00 ± 0.10‰, 2 SD)^1^. The positive Δ^199^Hg values indicate recycling of marine Hg into the asthenospheric mantle. Such a crustal Hg isotope signature was not observed in our samples of ocean island basalts and continental flood basalts, but has recently been identified in canonical end-member samples of the deep mantle^1^, therefore demonstrating that recycling of mercury can affect both the upper and lower mantle. Our study reveals large-scale translithospheric Hg recycling via plate tectonics.

## Abstract (web summary)

This work demonstrates distinct mass-independent mercury isotope fractionation in global basalts, which suggests mantle Hg isotope heterogeneity and reveals large-scale translithospheric Hg recycling via plate tectonics.

## Introduction

Mercury (Hg) is a unique heavy metal that has an active redox chemistry, high volatility, strong bioaccumulation, and extreme toxicity^2^. Both natural and anthropogenic activities emit large amounts of Hg to the atmosphere, mainly in the form of elemental Hg (Hg^0^)^3^. Gaseous Hg^0^ has a lifetime of ~1 year in the atmosphere, allowing its global transport before deposition into terrestrial and oceanic ecosystems via both wet and dry deposition pathways^4^. Due to its toxicity to humans and wildlife, the geochemical cycle of Hg in Earth’s surface environment (atmosphere, hydrosphere, and biosphere) has been extensively studied^2^. However, the cycling of Hg in Earth’s interior reservoirs (e.g., crust and mantle) remains less studied^1,5^.

Mercury ore deposits are predominantly located in active continental margin settings, with two well-known mercuriferous belts—the Pacific rim metallogenic belt and the Alpine-Himalayan belt^4^, implying a causal link between Hg metallogenesis and plate subduction. Mercury in oceanic reservoirs (e.g., sediments, seawater) may be carried by subducting ocean slabs into the asthenospheric mantle, and can then be emitted during arc magmatism to form Hg-enriched hydrothermal ore deposits in active continental margin settings^6^. A recent study, based on Hg isotopes in ocean island basalts, suggests that crustal Hg is also recycled into the lower mantle^1^.

Mercury is the only metal that exhibits both significant isotopic mass-dependent fractionation (MDF, defined as δ^202^Hg) and mass-independent fractionation (MIF, defined as Δ^199^Hg, Δ^200^Hg, Δ^201^Hg, and Δ^204^Hg)^7,8^. Hg-MDF is produced during essentially all biological, chemical, and physical processes involving Hg^7^. MIF of ^200^Hg and Δ^204^Hg is detected mainly in atmospherically derived samples, and values in rocks are generally too small to enable source tracing^8^. MIF of ^199^Hg and ^201^Hg mainly occurs during photochemical processes with little contribution from other reactions, and therefore provides clear source constraints^7^. On Earth’s surface, photochemical processes result in negative Δ^199^Hg (−0.6 to 0‰) in terrestrial reservoirs (e.g., soil and vegetation) and positive Δ^199^Hg (0 to 0.4‰) in oceanic reservoirs (e.g., seawater and marine sediments)^7,8^. It has been estimated that the primitive mantle has near-zero Δ^199^Hg (Δ^199^Hg: 0.00 ± 0.10‰, 2 SD) and negative δ^202^Hg (−1.7 ± 1.2‰, 2 SD), based on Hg isotope analyses of ^3^He-rich lavas^1^. A recent study also found significantly positive Δ^199^Hg (0 to 0.4‰) in hydrothermal ore deposits associated with arc magmatism, implying the recycling of Hg from marine sediments into arc lavas^6^.

Distinct Sr-Nd-Pb isotopic compositions of mid-ocean ridge basalts (MORBs), island arc basalts (IABs), ocean island basalts (OIBs), and continental flood basalts (CFBs) demonstrate a pronounced mantle isotope heterogeneity, which is generally accepted to result from recycling of crustal materials into the mantle via plate subduction^9,10^. MORBs are derived from partial melting of the upper mantle during the ascent of the asthenosphere beneath mid-ocean ridges. IABs result from partial melting of the mantle wedge at convergent margins due to the addition of subducted crustal materials (including volatile components and/or melts). OIBs and CFBs are genetically related to mantle plumes, which originate from the lower mantle or the core-mantle boundary^11^. A landmark study recently observed deviations in Δ^199^Hg in three well-characterized mantle endmembers (EM-1: −0.45 to 0.05‰; EM-2: 0.23‰; HIMU: −0.14‰), compared to the estimate of the primitive mantle (Δ^199^Hg: 0 ± 0.1‰, 2 SD) based on Hg isotope analyses of ^3^He-rich lavas, highlighting the great potential of using Hg isotopes to trace crustal Hg recycling into the mantle^1^.

**Fig. 1:**
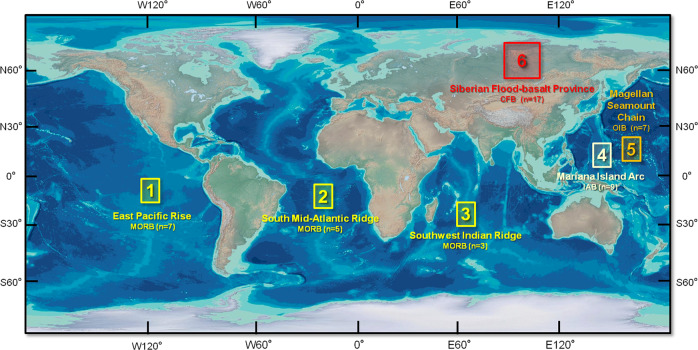
Geographic distribution of the sampling sites, sample type, and number of samples studied. Ten MORB (mid-ocean ridge basalt) samples are from the East Pacific Rise (1), Southern Mid-Atlantic Ridge (2), and Southwest Indian Ridge (3); nine IAB (island-arc basalt) samples are from the southern part of the Mariana Island Arc (4); seven OIB (ocean-island basalt) samples are from the Pako guyot of the Magellan Seamount Chain (5); and 17 CFB (continental flood basalt) samples are from the end-Permian Siberian Traps (6). The global map is modified after the World ocean bathymetric map by worldinmaps.com.

To aid in understanding the deep Hg cycle and to probe possible mantle Hg isotope heterogeneity, we measured the Hg isotopic compositions of a variety of types of basalts at a global scale (Fig. 1). Samples include MORBs from the Mid-Atlantic Ridge (MAR, *n* = 5), Southwest Indian Ridge (SWIR, *n* = 3), and East Pacific Rise (EPR, *n* = 7); IABs (*n* = 9) from the southern part of the Mariana Island Arc; HIMU-like OIBs (*n* = 2) and EM1-like OIBs (*n* = 5) from the Pako guyot of the Magellan Seamount Chain in the West Pacific Seamount Province; and CFBs (*n* = 17) from the Siberian Trap, the world’s largest flood basalt province. The geological background of these samples and analytical methods are given in the Materials and Methods section in detail. The sample information and analytical results are summarized in Supplementary Tables 1 and 2. We observed diverse Hg-MIF signals in these basalts, providing evidence of oceanic Hg recycling into the mantle.

## Results and discussion

### Hg concentrations and mass-dependent fractionation of Hg isotopes

**Fig. 2:**
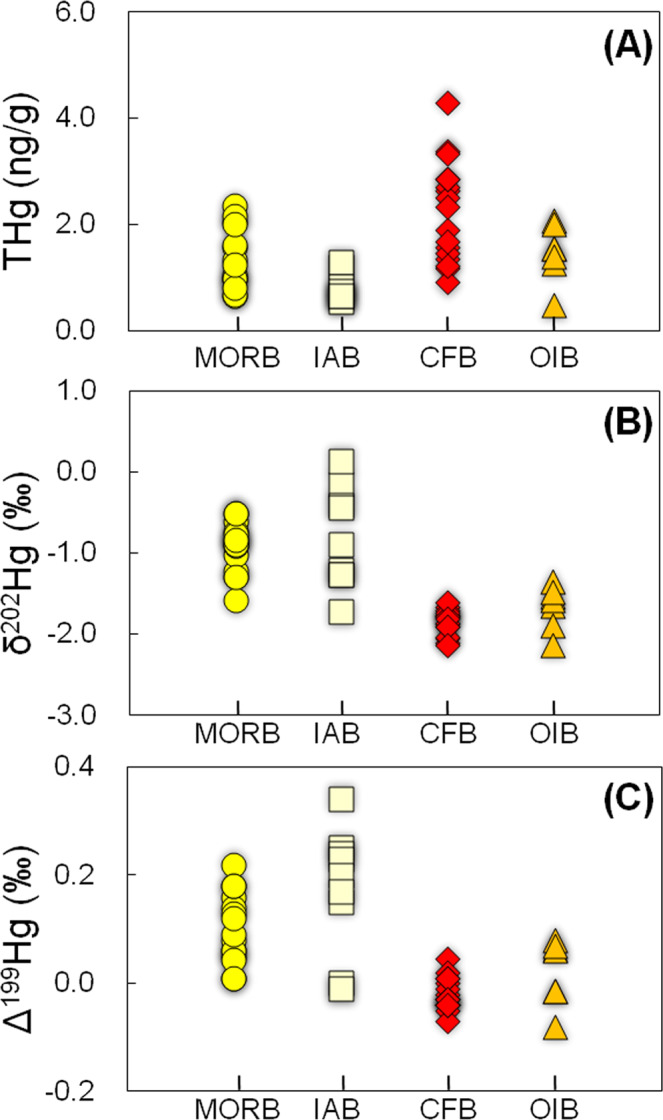
Total mercury content (THg) and mercury isotope data for the studied sample groups. Values for **A** THg, **B** δ^202^Hg, and **C** Δ^199^Hg in mid-ocean ridge basalts (MORB), island arc basalts (IAB), continental flood basalts (CFB), and ocean island basalts (OIB) were investigated in this study. Analytical uncertainties (2 SD) are 0.11‰ for δ^202^Hg and 0.07‰ for Δ^199^Hg. Analytical uncertainties (RSD) for THg are <9%.

In this study, total Hg (THg) concentrations vary from 0.65 to 2.35 ng/g for MORBs, from 0.54 to 1.28 ng/g for IABs, from 0.48 to 2.08 ng/g for OIBs, and from 0.91 to 4.29 ng/g for CFBs, with mean values of 1.48 ng/g, 2.25 ng/g, 1.63 ng/g, and 0.80 ng/g, respectively (Fig. 2A). These concentrations at the low ng/g level are in the range of mafic-ultramafic rocks worldwide (i.e., 0.2–7.0 ng/g for basalts and peridotite)^12,13^.

A large variation of δ^202^Hg was observed for all basalts studied (−2.13 to 0.13‰, Fig. 2B), which is ~20 times larger than the 2 SD analytical uncertainty for δ^202^Hg (±0.11‰). Specifically, MORBs and IABs have δ^202^Hg ranging from −1.58 to −0.50‰ and −1.72 to 0.13‰, with similar mean values of −0.93 ± 0.62‰ (2 SD) and −0.80 ± 1.22‰ (2 SD), respectively. OIBs and CFBs have relatively lower δ^202^Hg values of -2.13 to −1.60‰ and −2.13 to −1.48‰, with mean values of −1.85 ± 0.30‰ (2 SD) and −1.66 ± 0.54‰ (2 SD), respectively.

The large variation of δ^202^Hg in the studied basalts could be caused by MDF during magmatic processes. Lighter Hg isotopes may be preferentially volatilized during Hg degassing^14^, and thus generate enrichment of heavy Hg isotopes in the residue phases. The lower δ^202^Hg of fumarolic gas (−1.74 ± 0.36‰, 2 SD) compared to condensed particles (−0.74 ± 0.18‰, 2 SD) in an active volcano (Vulcano Island, Italy) strongly supports this view^14^. In our study, the more negative values of δ^202^Hg in OIBs and CFBs (that are genetically related to the lower mantle) relative to those of MORBs and IABs (that originate from the upper mantle), as shown in Fig. 2B, could possibly be explained by more severe Hg degassing during the eruption of the latter compared to the former.

An alternative explanation for the variation of δ^202^Hg in the basalts studied is the mixing of Hg sources with distinct δ^202^Hg values. The continental crust, in particular sedimentary rocks and sediments, displays large variations of δ^202^Hg (−3 to 1‰), with a mean value of −0.7 ± 1.6‰ (2 SD) (reviewed by Blum et al.^7^). The large variations of δ^202^Hg in MORBs and IABs in our study, coupled with their higher mean δ^202^Hg values compared to OIBs and CFBs, may be explained by the recycling of crustal rocks into the upper mantle. However, many different processes could trigger Hg-MDF^7^, which hinders the full understanding of Hg-MDF during magmatic processes. Thus, δ^202^Hg is not considered to be highly diagnostic of Hg sources. Instead, we focus on the Hg-MIF signal as a more reliable source tracer, as discussed below.

### Mass independent fractionation of Hg isotopes

**Fig. 3:**
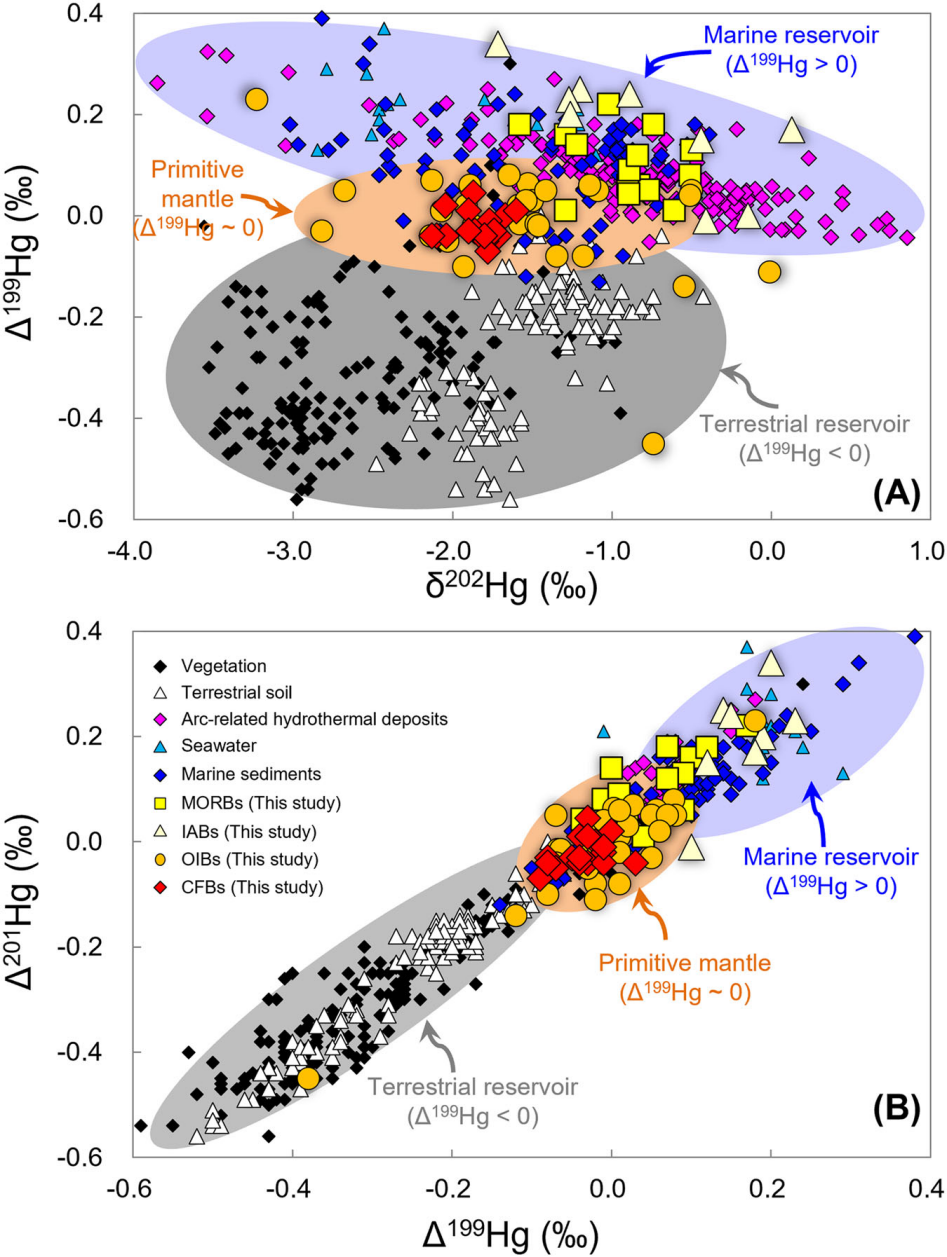
Mercury isotope correlation diagrams discriminate between terrestrial and marine Hg reservoirs and identify biogeochemical pathways of Hg isotope fractionation. **A** δ^202^Hg versus Δ^199^Hg diagram for mid-ocean ridge basalts (MORB, this study), island arc basalts (IAB, this study), continental flood basalts (CFB, this study), and ocean island basalts (OIB, this study and Moynier et al^1^.), compared with reference data for soil^16,19–21^, vegetation^16–18^, seawater^22^, marine sediments^23,24^, arc-related low-temperature hydrothermal deposits (Deng et al^6^. and references therein). The gray shaded area corresponds to the terrestrial reservoir (soil and vegetation) with negative Δ^199^Hg. The light blue shaded area represents the complementary marine reservoir (marine sediments and seawater) with positive Δ^199^Hg. The light orange shaded area represents the primitive mantle with Δ^199^Hg ≈ 0. **B** Δ^201^Hg versus Δ^199^Hg diagram. The correlation trend with a slope of 1 identifies photochemical reduction of Hg (II) as the process which controls mass-independent fractionation of mercury (Blum et al^7^.). Analytical uncertainties (2 SD) for the data are 0.11‰ for δ^202^Hg and 0.07‰ for Δ^199^Hg and Δ^201^Hg.

All samples investigated show limited or no MIF of ^200^Hg, with Δ^200^Hg ranging from −0.08 to 0.08‰, which is nearly within the analytical uncertainty for Δ^200^Hg (±0.06‰). However, large variations of Δ^199^Hg (−0.08 to 0.34‰) and Δ^201^Hg (−0.09 to 0.23‰) are observed in the samples studied, compared to the analytical uncertainties for Δ^199^Hg (±0.07‰) and Δ^201^Hg (±0.06‰). MIF of ^199^Hg and ^201^Hg is mainly produced during aqueous Hg(II) photoreduction on Earth’s surface with little contribution from other processes^7,15^. Magmatic degassing could produce limited Hg-MIF of <0.1‰ in Δ^199^Hg and Δ^201^Hg^5^. Hg(II) photoreduction imparts negative Δ^199^Hg in the product gaseous Hg(0) and positive Δ^199^Hg in the residual Hg(II) phase^15^, with Δ^199^Hg/Δ^201^Hg of ~1. For this reason, terrestrial vegetation mostly shows negative Δ^199^Hg (−0.6 to 0‰, Fig. 3A), due to the primary accumulation of Hg(0) during uptake by foliage^16–18^. Soil receives a substantial amount of Hg from litterfall^16,19–21^, and thus is characterized by negative Δ^199^Hg (−0.6 to 0‰, Fig. 3A). In comparison, the oceans mainly receive Hg through wet deposition of Hg(II), resulting in positive Δ^199^Hg in seawater (0 to 0.4‰) and marine sediments^22–24^ (−0.1 to 0.4‰, Fig. 3A). Terrestrial and marine reservoirs both show Δ^199^Hg/Δ^201^Hg of ~1 (Fig. 3B), suggesting that Hg-MIF is mainly caused by Hg(II) photoreduction.

### Crustal Hg recycling into the lower mantle

OIBs and CFBs are petrogenetically related to the lower mantle. As shown in Fig. 3A, OIBs and CFBs investigated in this study show negative δ^202^Hg (−1.80 ± 0.42‰, 2 SD) and near-zero Δ^199^Hg values (−0.01 ± 0.08‰, 2 SD), which are within the range of the estimate of the primitive mantle (δ^202^Hg: −1.7 ± 1.2‰; Δ^199^Hg: 0 ± 0.1‰) based on Hg isotope analyses of ^3^He-rich lavas^1^. The recently observed significant Δ^199^Hg signals in two EM-1 OIBs (−0.45 and −0.11‰), an EM-2 OIB (0.23‰), and a HIMU OIB (−0.14‰)^1^, imply that Hg in oceanic and continental crustal materials can be recycled into the lower mantle. However, the amount of recycled Hg is likely small with respect to the total Hg pool in the lower mantle, given the fact that OIBs and CFBs in this study as well as 16 previously studied OIBs (including 11 ^3^He-rich lavas)^1^ show near-zero Δ^199^Hg values (Fig. 3A).

### Recycling of oceanic Hg into the upper mantle

**Fig. 4:**
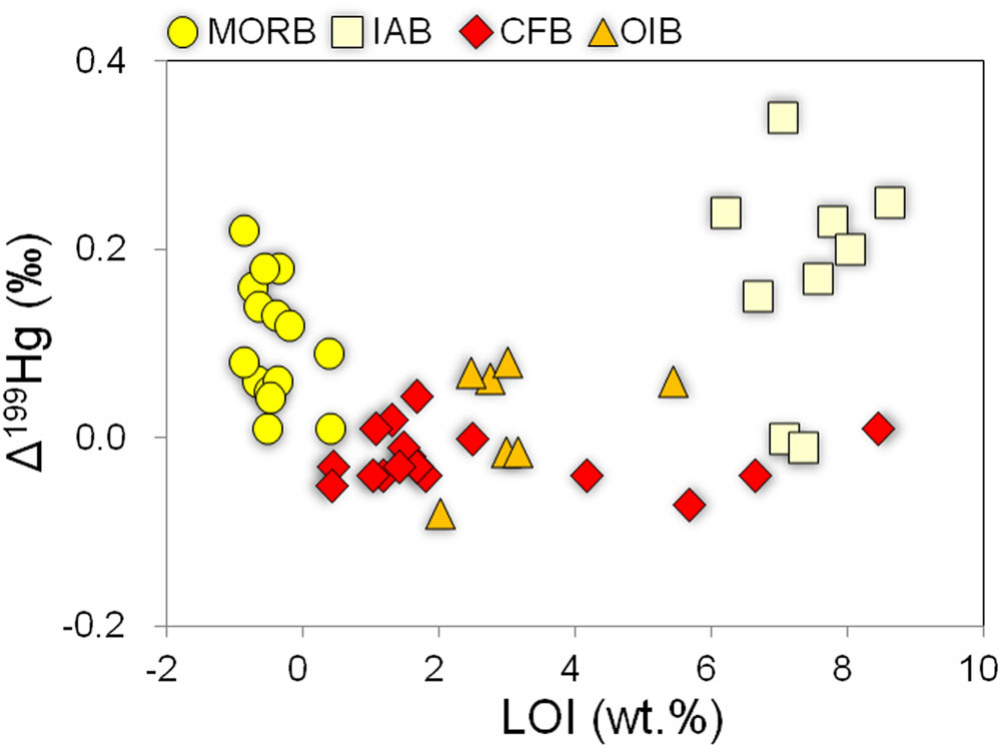
Loss on ignition (LOI, wt%) as a measure of alteration versus Δ^199^Hg in mid-ocean ridge basalts (MORB), island arc basalts (IAB), continental flood basalts (CFB), and ocean island basalts (OIB) investigated in this study. The lack of correlation between secondary alteration and Δ^199^Hg in the studied samples suggests no significant disturbance of the Δ^199^Hg values by external fluids. Analytical uncertainties (2 SD) are 0.07‰ for Δ^199^Hg. Analytical uncertainties (RSD) for LOI are <5%.

MORBs and IABs have mostly positive Δ^199^Hg values of 0.05 to 0.22‰ and -0.01 to 0.34‰ (Figs. 2C, 3A), with Δ^199^Hg/Δ^201^Hg of ~1 (Fig. 3B). These values fall within the range of marine sediments and seawater, suggesting that a substantial amount of Hg in these rocks is of marine origin. Their positive Δ^199^Hg values may result from the fixation of seawater Hg during seawater-rock reactions. MORBs and IABs investigated here were collected from the seafloor and show variable weight loss on ignition (LOI) ranging from −0.9 to 0.4 wt% and 6.2 to 8.0 wt%, respectively (Supplementary Table 2). The very low LOI of the MORBs and their pristine chemical and mineralogical composition argue against seawater overprinting (Supplementary Table 2). Although IABs have high LOI, no correlations are observed between LOI and THg or Δ^199^Hg (Fig. 4), indicating that seawater-rock reactions are unlikely to be the mechanism responsible for the positive Δ^199^Hg in IABs. The OIB samples studied here were also collected from the seafloor. Although these OIBs have relatively higher LOI values (2.0 to 8.6 wt%, Supplementary Table 2) than MORBs, they have near-zero Δ^199^Hg values. Given the low Hg concentration in seawater (<0.02 to 2 ng/L)^25^, seawater-rock reactions can be further precluded as a reason for the positive Δ^199^Hg values in MORBs and IABs.

We attribute the positive Δ^199^Hg signals in the studied IABs to recycled marine sediments. At convergent margins, subduction of the oceanic slab carries a substantial amount of Hg from marine sediments into subduction zones. The downgoing slab undergoes dehydration and metamorphism and releases fluids and melts that in turn metasomatize the mantle wedge. Partial melting of such metasomatized mantle could generate IABs with positive Δ^199^Hg. Notably, given the low volatilization temperature of Hg, subducted Hg may be mostly released from the downgoing slab and returned to the Earth’s surface through arc volcanism. This is supported by the positive Δ^199^Hg signal observed in volcanic arc-related epithermal Hg-Au deposits in NE China and the Pacific rim (0 to 0.4‰, Fig. 3A, B, Deng et al.^6^ and references therein). The most recent estimate of present-day global volcanic Hg flux (76 ± 30 t/year)^26^ is slightly lower than the total mass of Hg in global subducted sediments (~87.4 t/year), calculated from the mass of global subducted sediment (1.4 × 10^15 ^g/year)^27^ multiplied by the Hg concentration of average marine sediments (62.4 ng/g)^28^.

The positive Δ^199^Hg of MORBs could also be the result of the recycling of oceanic crust. In subduction zones, the subducted marine Hg may not be completely released via arc volcanism. The remaining Hg in the subducted slab could continue to descend into the asthenospheric mantle. This fraction of Hg is recycled into the asthenosphere and could be brought back to Earth’s surface via basaltic magmatism at mid-ocean ridges. The upper mantle is heterogeneous in Hg isotopes, apparently due to the variable involvement of recycled crustal Hg. Such isotopic heterogeneities are consistent with large variations in Sr-Nd-Pb isotope compositions for MORBs from different mid-ocean ridges (e.g., MAR, SWIR, and EPR), which supports the model of recycling of crustal materials into the upper mantle^29–31^. The recycling of oceanic Hg into the upper mantle is also favored by the positive Δ^199^Hg values (0.07 to 0.27‰) observed in hotspring and sinter samples from the Yellowstone Plateau volcanic field^32^, which might be explained by upwelling asthenospheric mantle flow induced by the subducting Pacific plate^33^.

### Geodynamic implications

**Fig. 5:**
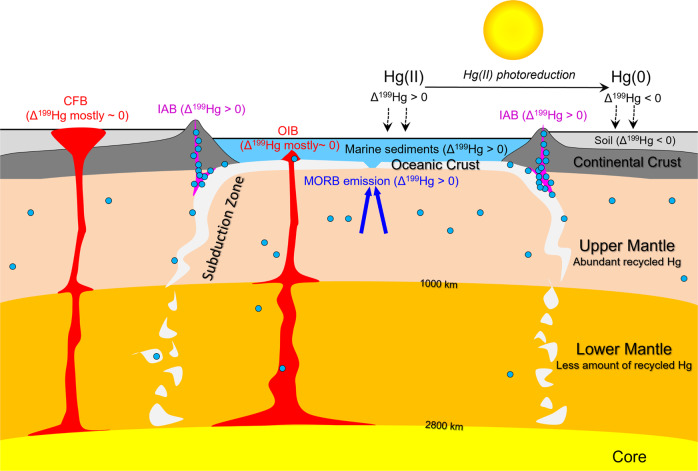
Conceptual model showing the deep cycling of mercury on Earth (not to scale). Photoreduction of Hg(II) produces gaseous Hg(0) with negative Δ^199^Hg values and gaseous Hg(II) with positive Δ^199^Hg values. Gaseous Hg (0) is preferentially accumulated in soil and vegetation (light gray area), whereas gaseous Hg(II) is readily deposited into the marine reservoir (light blue area). In subduction zones, oceanic crust (white-gray area) is subducted underneath the continental crust (dark gray area) and delivers large amounts of Hg from marine sediments (light blue circles) into the mantle. Most of the subducted Hg is released and cycled back to the surface environment through arc volcanism, forming island arc basalts (IAB, purple area) with positive Δ^199^Hg values. The remaining Hg in the subducting slab is cycled into the upper mantle (light orange area) and released at mid-ocean ridges by mid-ocean ridge basalt volcanism (MORB, highlighted in blue), which displays positive Δ^199^Hg values. Some of the subducted Hg may also be cycled into the lower mantle (orange area), resulting in Δ^199^Hg signals in some ocean island basalts (OIB)^1^; however, given that OIBs and CFBs mostly show near-zero Δ^199^Hg values (highlighted in red), this recycled Hg may represent only a small proportion of Hg in the lower mantle.

Based on our current knowledge of Hg isotopic variation in mantle-derived rocks, the deep cycling of Hg is illustrated in Fig. 5. Photochemical reactions produce significant negative Δ^199^Hg values in terrestrial reservoirs (e.g., soil and vegetation) and significant positive Δ^199^Hg values in marine reservoirs (e.g., seawater and marine sediments). Mercury from terrestrial and marine reservoirs is subducted at convergent margins. Due to the low volatilization temperature of Hg, much of the subducted Hg is likely released back to the surface environment through arc volcanism, forming IABs and arc-related epithermal Hg deposits with significantly positive Δ^199^Hg values. The remaining Hg in the subducted slab is recycled into the asthenosphere and released at mid-ocean ridges by MORBs, which are characterized by significantly positive Δ^199^Hg values.

Some of the subducted Hg may also be recycled into the lower mantle, consistent with the recently observed significant Δ^199^Hg signals in some OIB samples^1^; however, given that most OIBs and CFBs show near-zero Δ^199^Hg values, this recycled Hg likely represents only a small proportion of Hg in the lower mantle, and may be mostly located in the source of the mantle end-members. In the future, analysis of OIBs and CFBs from additional sites will be needed to better quantified the amount of recycled Hg in the lower mantle.

Overall, our study demonstrates mantle Hg isotopic heterogeneity and reveals large-scale translithospheric Hg recycling via plate tectonics. Hg tends to be depleted in volcanic rocks derived from the mantle (e.g., several ng/g in basalts) and enriched in crustal sedimentary rocks (e.g., 62.4 ng/g in average marine sediments^28^). Given the significantly large Δ^199^Hg values and high Hg concentrations in crustal materials, we infer that the crustal signature cannot easily be erased by the primitive mantle signature (Δ^199^Hg ~ 0). We conclude that Hg-MIF (Δ^199^Hg) can be a useful tool for studying crust-mantle interactions.

## Methods

### Samples and geological background

The sample locations are shown in Fig. 1 and summarized in Supplementary Table 1. MORBs were collected from the EPR (*n* = 7), MAR (*n* = 5), and SWIR (*n* = 3), during the DY115-21, DY115-22, DY125-30, and DY135-40 cruises, by the Chinese R/V DayangYihao using TV grab (grab system controlled via a TV camera). The three regions have different spreading rates (EPR: 80 mm/yr; MAR: 35 mm/yr; SWIR: 14 mm/yr), representing a broad spectrum of crustal accretion modes^34,35^.

IABs (*n* = 9) were collected from the southern part of the Mariana Island Arc, using the submersible Jiaolong, during the R/V Xiangyanghong 09 Dayang 37th cruise. The Mariana Island Arc is a classic young island arc in the western Pacific Ocean. Most of the islands and underwater volcanoes in the southern part of the Mariana Island Arc were formed during the Eocene to Miocene^36^.

The Pako guyot was formed by the large-scale eruption of a hotspot at 120–90 Ma^37^. Seven OIBs were collected from the Pako guyot of the Magellan Seamount Chain in the West Pacific Seamount Province (Fig. 1), during Chinese R/V Dayang Yihao and Xiangyanghong 09 cruises (DY105 and DY31), using the submersible Jiaolong^38^. Two samples (JL-Dive80-ST01-S01-1 and JL-Dive80-ST03-S05-1) show HIMU-like Sr-Nd-Pb-Hf isotopic compositions indicating recycled ancient oceanic crust in their mantle source, while the others display EM1-like isotopic compositions consistent with recycled sub-continental lithospheric components in their mantle source.

CFBs (*n* = 17) were sampled from the Norilsk region at the northwestern margin of the Siberian platform, which is a part of the world’s largest flood basalt province, the Siberian Traps (Fig. 1). The eruption of these CFBs occurred from the latest Permian to the Early Triassic (i.e., 248–252 Ma) and was associated with the break-up of Gondwanaland^39^. Trace element and Sr-Nd isotope data indicate that CFBs from the Norilsk region were formed by the interaction of plume-generated picritic magmas with the lithosphere^40^.

### Chemical analyses

The samples were cut to expose fresh surfaces, washed by 18.2 MΩ cm water, air-dried, powdered, and homogenized, prior to chemical analysis at the Institute of Geochemistry, Chinese Academy of Sciences (IGCAS). Major elements were analyzed at the ALS Minerals-ALS Chemex, Guangzhou, China, using a PANalytical PW2424 X-ray fluorescence spectrometer. LOI values were measured by combustion loss of weight, with analytical uncertainties (RSD) of <5%. Total Hg (THg) concentration was determined by an RA-915+ Hg analyzer (Lumex, Russia), with a detection limit of 0.01 ng/g. Standard reference materials (GSR-2, andesite; BCR-2, basalt) were measured, yielding Hg recoveries of 94–106% and RSD of <9%.

The samples were prepared for Hg isotope analysis using a double-stage tube furnace coupled with 40% anti aqua regia (HNO_3_/HCl = 2/1, v/v) trapping solutions^41^. Standard reference materials (GSR-2 and BCR-2) and method blanks were processed in the same way as the samples. The former yielded Hg recoveries of 90–105% and the latter showed Hg concentrations lower than the detection limit, precluding lab contamination. The preconcentrated solutions were diluted to 0.5 ng/mL with an acid concentration of 10–20% prior to Hg isotope analysis using a Neptune Plus multi-collector inductively coupled plasma mass spectrometry^42^. Hg isotope ratios were reported following the convention proposed by Blum and Bergquist^43^. MDF is expressed in δ^202^Hg notation in units of ‰ referenced to the NIST-3133 (analyzed before and after each sample):

1 $${\delta}^{{{{{\rm{202}}}}}}{{{{{\mathrm{Hg}}}}}}(\permil)=[({\!\,}^{202} {{{{{\mathrm{Hg}}}}}}{/}{\!\,}^{198} {{{{{\mathrm{Hg}}}}}}_{{{{{\mathrm{sample}}}}}}){/} ({{\!\,}^{202}} {{{{{\mathrm{Hg}}}}}}{/} {\!\,}^{198} {{{{{\mathrm{Hg}}}}}}_{{{{{\mathrm{standard}}}}}})-1]\times 1000$$

MIF is reported in Δ notation, which describes the difference between the measured δ^xxx^Hg and the theoretically predicted δ^xxx^Hg value, in units of ‰:

2 $${\Delta }^{{{{{\rm{xxx}}}}}}{{{{{\mathrm{Hg}}}}}}={{\delta }}^{{{{{\mathrm{xxx}}}}}}{{{{{\mathrm{Hg}}}}}}-{{\delta }}^{202}{{{{{\mathrm{Hg}}}}}}\times\beta$$

β is 0.252 for ^199^Hg, 0.5024 for ^200^Hg, and 0.752 for ^201^Hg. Hg concentration and acid matrices in the bracketing NIST-3133 solutions were matched with neighboring samples. NIST-3177 secondary standard solutions, diluted to 0.5 ng/mL Hg with 10% HCl, were measured every 10 samples. The overall average and uncertainty of NIST-3177 (δ^202^Hg: -0.53 ± 0.10‰; Δ^199^Hg: -0.03 ± 0.04‰; Δ^200^Hg: 0.00 ± 0.05‰; Δ^201^Hg: -0.02 ± 0.04‰; 2 SD, *n* = 26) and GSR-2 (δ^202^Hg: -1.62 ± 0.11‰; Δ^199^Hg: 0.04 ± 0.06‰; Δ^200^Hg: 0.01 ± 0.04‰; Δ^201^Hg: 0.02 ± 0.06‰, 2 SD, *n* = 5) and BCR-2 (δ^202^Hg: -1.89 ± 0.11‰; Δ^199^Hg: 0.00 ± 0.07‰; Δ^200^Hg: 0.00 ± 0.06‰; Δ^201^Hg: 0.01 ± 0.06‰, 2 SD, *n* = 5) agree well with previous results^43–45^. The larger of the values of standard deviation (2 SD) for either NIST-3177, GSR-2, or BCR-2 are used to reflect maximum analytical uncertainties.

## Supplementary information

Supplementary Information

## Data Availability

All data is available in the main text or supplementary materials.
