# Supplementary material for: Mantle Hg isotopic heterogeneity and evidence of oceanic Hg recycling into the mantle
Source: Nat Commun. 2022 Feb 17;13:948. doi: 10.1038/s41467-022-28577-1 (PMC8854601; doi:10.1038/s41467-022-28577-1)
Supplement: Supplementary file 1 — Supplementary Information [file 41467_2022_28577_MOESM1_ESM.pdf]

Supplementary Information for

Mantle Hg isotopic heterogeneity and evidence of oceanic Hg recycling into the mantle

Runsheng Yin, Di Chen, Xin Pan, Changzhou Deng, Liemeng Chen, Xieyan Song, Songyue Yu,

Chuanwei Zhu, Xun Wei, Yue Xu, Xinbin Feng, Joel D. Blum, Bernd Lehmann

Supplementary Table 1 Sample information, Hg concentration and Hg isotopic composition.

Sample	Location	Longitude	Latitude	Depth	Type	THg	δ^{202}Hg	Δ^{199}Hg	Δ^{200}Hg	Δ^{201}Hg
ID						ppb	‰	‰	‰	‰
22VI-EPR-S003-TVG01	East Pacific Rise	102.553	3.103	2905	MORB	0.96	-1.29	0.16	0.00	0.10
22 VI -EPR-S005-TVG02	East Pacific Rise	102.704	2.650	2915	MORB	2.35	-1.22	0.14	0.03	0.00
22 VI -EPR-S007-TVG04	East Pacific Rise	102.532	3.007	2925	MORB	1.06	-0.83	0.06	-0.03	0.08
22 VI -EPR-S0014-TVG09	East Pacific Rise	102.552	3.110	2888	MORB	1.58	-0.61	0.01	0.04	0.04
22 VI -EPR-S0024-TVG14	East Pacific Rise	102.551	3.103	2895	MORB	0.98	-1.02	0.22	0.05	0.17
22 VI -EPR-S0021-TVG15	East Pacific Rise	102.554	3.099	2914	MORB	2.05	-0.74	0.18	0.00	0.07
22 VI -EPR-S0024-TVG18	East Pacific Rise	102.552	3.103	2906	MORB	0.65	-0.51	0.08	-0.01	-0.01
22V-SMAR-S017-TVG02	Mid-Atlantic Ridge	13.489	14.725	2835	MORB	1.34	-0.77	0.05	-0.08	0.02
22 V -SMAR-S025-TVG06	Mid-Atlantic Ridge	13.336	15.207	3088	MORB	1.62	-0.9	0.06	0.05	0.09
561-2-2 (67-70)	Mid-Atlantic Ridge			3459	MORB	0.98	-0.89	0.09	0.02	0.01
563-25-1 (47-50)	Mid-Atlantic Ridge	13.489	14.725	2835	MORB	0.68	-0.89	0.04	0.01	-0.04
563-1-1 (9-12)	Mid-Atlantic Ridge	13.336	15.166	2772	MORB	0.81	-1.58	0.18	-0.01	0.12
40II-TVG14	Southwest Indian Ridge	49.258	-37.943		MORB	2.17	-1.29	0.01	0.03	-0.02
30III-TVG12	Southwest Indian Ridge	50.473	-37.661	1813	MORB	1.24	-0.5	0.13	-0.02	0.09
40I-S002-TVG01	Southwest Indian Ridge	50.467	-37.658		MORB	2	-0.84	0.12	0.02	0.07
mar-JL143-S02	Mariana Island Arc	141.967	11.819	4809	IAB	1.21	-0.15	0.00	0.02	0.00
mar-JL143-S03	Mariana Island Arc	141.967	11.819	4809	IAB	1.28	-0.41	-0.01	0.01	0.1
mar-JL143-S05	Mariana Island Arc	141.967	11.818	4792	IAB	0.68	-1.20	0.25	0.03	0.14
mar-JL143-S06	Mariana Island Arc	141.967	11.818	4792	IAB	0.62	-1.72	0.34	0.08	0.20
mar-JL143-S09	Mariana Island Arc	141.967	11.819	4809	IAB	0.83	-0.89	0.24	0.07	0.15
mar-JL143-S10	Mariana Island Arc	141.962	11.817	4723	IAB	0.54	-0.44	0.15	0.08	0.12
mar-JL143-S11	Mariana Island Arc	141.956	11.812	4704	IAB	0.65	-1.27	0.23	0.04	0.23
mar-JL143-S12	Mariana Island Arc	141.955	11.812	4699	IAB	0.74	-1.26	0.2	0.05	0.19
mar-JL143-wz	Mariana Island Arc	141.956	11.812	4702	IAB	0.65	0.13	0.17	0.06	0.18
JL-Dive80-ST01-S01-1	Pako guyot	155.290	15.980	3145.4	OIB	1.56	-1.53	0.06	0.00	0.00

JL-Dive80-ST03-S05-1	Pako guyot	155.280	15.980	2719	OIB	2.08	-1.65	0.08	0.02	0.08
MASD10‡	Pako guyot	154.950	15.490	1493	OIB	1.26	-1.35	-0.08	-0.04	-0.02
JL-Dive75-ST01-S01-1	Pako guyot	155.310	15.540	1534	OIB	1.56	-1.58	-0.01	0.02	-0.07
JL-Dive75-ST02-S02-1	Pako guyot	155.310	15.540	1534	OIB	0.48	-1.48	-0.02	0.02	-0.06
JL-Dive80-ST01-S03-2	Pako guyot	155.290	15.980	3145.4	OIB	2.01	-1.89	0.06	-0.05	0.06
JL-Dive81-ST02-S03-1	Pako guyot	154.920	15.680	1836.7	OIB	1.39	-2.13	0.07	0.05	0.03
RN-1	Norilsk region, Siberian	69.6176	88.2975		CFB	2.64	-1.67	0.00	0.06	-0.02
RN-2	Norilsk region, Siberian	69.6185	88.3127		CFB	2.7	-1.78	-0.02	0.02	-0.01
RN-3	Norilsk region, Siberian	69.6187	88.3177		CFB	2.51	-2.1	-0.04	0.01	-0.03
RN-4	Norilsk region, Siberian	69.6186	88.3214		CFB	1.58	-1.87	0.05	0.03	-0.03
RN-5	Norilsk region, Siberian	69.6194	88.3238		CFB	1.36	-2.05	0.02	0.00	0.00
RN-6	Norilsk region, Siberian	69.6194	88.3300		CFB	1.18	-2.04	-0.04	0.02	0.03
RN-7	Norilsk region, Siberian	69.6188	88.3366		CFB	3.38	-1.71	-0.04	-0.01	-0.01
RN-8	Norilsk region, Siberian	69.6189	88.3504		CFB	1.90	-1.77	-0.01	-0.02	-0.01
RN-9	Norilsk region, Siberian	69.6216	88.3712		CFB	0.91	-1.86	-0.03	0.03	-0.08
RN-10	Norilsk region, Siberian	69.6219	88.3730		CFB	1.46	-1.79	-0.05	0.06	-0.07
RN-11	Norilsk region, Siberian	69.6197	88.3642		CFB	2.33	-1.74	-0.03	0.01	-0.05
RN-14*	Norilsk region, Siberian	69.212	88.009	626.5 m	CFB	4.29	-1.9	0.01	-0.01	-0.04
RN-16*	Norilsk region, Siberian	69.212	88.009	629 m	CFB	2.85	-1.78	-0.07	-0.03	-0.09
RN-17*	Norilsk region, Siberian	69.212	88.009	636m	CFB	2.85	-2.13	-0.04	0.03	-0.08
RN-19*	Norilsk region, Siberian	69.212	88.009	762 m	CFB	1.69	-1.6	0.01	-0.01	-0.03
RN-21	Norilsk region, Siberian	69.2448	88.2390		CFB	1.23	-1.9	-0.03	0.03	-0.04
RN-22	Norilsk region, Siberian	69.2686	88.1149		CFB	3.33	-1.8	-0.04	0.02	-0.04

*These samples were collected from the drill core OM-3 that cut through the lower volcanic suites of the Siberian flood basalts.

Supplementary Table 2 Major element data

Sample	SiO_2	Al_2O_3	TFe_2O_3	MgO	CaO	Na_2O	K_2O	MnO	P_2O_5	TiO_2	LOI	Total
ID	%	%	%	%	%	%	%	%	%	%	%	%
22VI-EPR-S003-TVG01	51.1	13.3	13.0	6.57	10.52	2.91	0.10	0.20	0.17	1.92	-0.7	99.1
22 VI -EPR-S005-TVG02	52.0	12.8	14.2	4.60	8.34	3.62	0.34	0.22	0.35	2.36	-0.7	98.2
22 VI -EPR-S007-TVG04	51.0	14.7	9.8	8.18	12.12	2.54	0.06	0.16	0.10	1.17	-0.7	99.1
22 VI -EPR-S0014-TVG09	50.9	13.8	11.7	7.03	11.15	2.87	0.11	0.19	0.15	1.66	-0.5	99.0
22 VI -EPR-S0024-TVG14	51.0	13.5	12.5	6.65	10.78	2.92	0.11	0.20	0.17	1.83	-0.9	98.7
22 VI -EPR-S0021-TVG15	51.1	13.9	11.0	7.42	11.54	2.76	0.09	0.18	0.12	1.42	-0.3	99.2
22 VI -EPR-S0024-TVG18	50.8	13.1	12.9	6.55	10.52	2.86	0.10	0.20	0.17	1.88	-0.9	98.2
22V-SMAR-S017-TVG02	49.1	15.5	10.4	7.69	11.51	2.75	0.14	0.16	0.14	1.43	-0.5	98.3
22 V -SMAR-S025-TVG06	49.3	15.1	10.8	8.09	11.76	2.64	0.10	0.17	0.13	1.40	-0.4	99.1
561-2-2 (67-70)	50.3	14.7	11.3	7.04	12.02	2.13	0.17	0.19	0.06	1.38	0.38	99.64
563-25-1 (47-50)	49.4	15.5	10.4	7.61	11.61	2.78	0.13	0.16	0.14	1.44	-0.5	98.7
563-1-1 (9-12)	49.5	15.4	10.6	7.99	11.29	2.91	0.29	0.16	0.21	1.61	-0.6	99.4
40II-TVG14	48.9	18.3	10.0	6.15	12.54	2.45	0.22	0.16	0.11	1.28	0.4	100.5
30III-TVG12	49.8	15.0	11.7	7.96	11.30	2.77	0.10	0.19	0.13	1.42	-0.4	100.0
40I-S002-TVG01	48.0	16.2	10.5	10.29	11.19	2.53	0.16	0.16	0.05	0.84	-0.2	99.6
mar-JL143-S02	47.5	17.6	10.1	3.36	5.65	3.54	1.49	1.65	0.23	0.77	7.1	99.0
mar-JL143-S03	48.4	18.2	9.3	3.40	5.35	3.77	1.60	0.74	0.23	0.76	7.3	99.1
mar-JL143-S05	47.6	18.3	9.4	3.39	4.79	3.79	1.66	1.03	0.20	0.78	8.6	99.6
mar-JL143-S06	48.1	18.3	9.8	3.36	5.57	3.83	1.65	0.60	0.25	0.80	7.0	99.4
mar-JL143-S09	49.6	19.2	8.2	3.37	6.06	3.90	1.66	0.36	0.26	0.74	6.2	99.5
mar-JL143-S10	49.3	17.9	9.0	3.41	4.68	3.91	2.08	0.66	0.29	0.79	6.7	98.7
mar-JL143-S11	48.9	18.1	8.8	3.62	4.80	3.83	1.73	0.53	0.25	0.74	7.8	99.1
mar-JL143-S12	48.6	17.4	9.3	3.64	4.49	3.87	1.84	0.82	0.27	0.74	8.0	98.9
mar-JL143-wz	47.5	17.9	9.8	3.34	5.58	3.71	1.52	1.46	0.25	0.79	7.6	99.4
JL-Dive80-ST01-S01-1	42.8	15.9	16.8	2.96	9.41	2.10	0.93	0.14	0.50	5.47	2.7	99.7

JL-Dive80-ST03-S05-1	43.2	14.6	19.0	2.24	8.02	2.46	1.33	0.19	0.86	5.15	3.0	100.1
MASD10[‡]	48.6	16.2	13.0	2.24	7.15	3.74	2.59	0.17	0.48	3.22	2.0	99.4
JL-Dive75-ST01-S01-1	47.5	17.9	8.6	2.83	8.64	2.71	2.57	0.09	2.14	3.82	3.0	99.76
JL-Dive75-ST02-S02-1	48.7	18.5	7.9	2.62	7.56	2.65	2.87	0.08	1.88	3.96	3.1	99.80
JL-Dive80-ST01-S03-2	45.1	15.8	15.5	2.41	6.59	3.57	1.46	0.09	0.69	3.49	5.4	100.0
JL-Dive81-ST02-S03-1	44.9	14.5	13.3	6.72	8.92	2.62	1.82	0.14	0.45	3.66	2.5	99.5
RN-1	49.7	15.3	10.5	7.33	7.30	2.63	2.82	0.19	0.14	1.04	2.5	99.4
RN-2	49.2	15.6	10.9	6.12	11.32	2.59	0.69	0.17	0.19	0.95	1.6	99.3
RN-3	49.2	14.7	11.6	7.40	11.16	2.30	0.60	0.18	0.13	1.08	1.2	99.5
RN-4	49.2	15.3	11.2	5.82	10.91	2.78	0.96	0.17	0.19	0.99	1.7	99.2
RN-5	49.2	14.7	11.8	6.99	11.37	2.36	0.43	0.17	0.13	1.09	1.3	99.5
RN-6	48.8	15.1	12.8	7.18	10.40	2.62	0.50	0.19	0.14	1.22	1.0	99.9
RN-7	47.5	14.9	12.7	7.47	10.38	2.44	0.47	0.18	0.12	1.10	1.8	99.0
RN-8	48.5	15.1	12.3	7.25	10.63	2.40	0.47	0.18	0.14	1.17	1.5	99.5
RN-9	48.4	14.9	13.4	6.97	10.37	2.55	0.43	0.20	0.15	1.40	0.4	99.2
RN-10	48.7	15.0	13.4	6.91	10.51	2.63	0.30	0.20	0.15	1.39	0.4	99.6
RN-11	48.1	14.9	12.8	7.14	10.84	2.44	0.16	0.20	0.15	1.28	1.7	99.6
RN-14	44.0	9.6	12.1	11.14	10.77	1.58	0.10	0.19	0.13	1.43	8.4	99.4
RN-16	47.5	11.0	11.7	9.03	10.72	1.73	0.18	0.16	0.13	1.46	5.7	99.3
RN-17	51.4	11.5	11.1	8.97	4.41	3.23	0.24	0.11	0.16	1.80	6.6	99.5
RN-19	52.0	14.0	13.2	3.57	6.80	3.50	2.28	0.19	0.76	2.39	1.1	99.6
RN-21	49.5	15.3	11.5	5.86	10.71	2.75	1.01	0.16	0.20	1.01	1.4	99.3
RN-22	44.0	13.4	15.6	3.67	8.24	3.30	2.17	0.22	1.44	3.68	4.2	99.8
